# Geographical separation and ethnic origin influence the human gut microbial composition: a meta-analysis from a Malaysian perspective

**DOI:** 10.1099/mgen.0.000619

**Published:** 2021-08-31

**Authors:** Jacky Dwiyanto, Qasim Ayub, Sui Mae Lee, Su Chern Foo, Chun Wie Chong, Sadequr Rahman

**Affiliations:** ^1^​ School of Science, Monash University Malaysia, Bandar Sunway, Malaysia; ^2^​ Monash University Malaysia Genomics Facility, Bandar Sunway, Malaysia; ^3^​ School of Pharmacy, Monash University Malaysia, Bandar Sunway, Malaysia; ^4^​ Institute for Research, Development and Innovation, International Medical University, Kuala Lumpur, Malaysia; ^5^​ Tropical Medicine and Biology Multidisciplinary Platform, Monash University Malaysia, Bandar Sunway, Malaysia

**Keywords:** community, Chinese, gut microbiome, Indian, microbiota, Malay, next-generation sequence

## Abstract

Ethnicity is consistently reported as a strong determinant of human gut microbiota. However, the bulk of these studies are from Western countries, where microbiota variations are mainly driven by relatively recent migration events. Malaysia is a multicultural society, but differences in gut microbiota persist across ethnicities. We hypothesized that migrant ethnic groups continue to share fundamental gut traits with the population in the country of origin due to shared cultural practices despite subsequent geographical separation. To test this hypothesis, the 16S rRNA gene amplicons from 16 studies comprising three major ethnic groups in Malaysia were analysed, covering 636 Chinese, 248 Indian and 123 Malay individuals from four countries (China, India, Indonesia and Malaysia). A confounder-adjusted permutational multivariate analysis of variance (PERMANOVA) detected a significant association between ethnicity and the gut microbiota (PERMANOVA *R* ^2^=0.005, pseudo-*F*=2.643, *P*=0.001). A sparse partial least squares – discriminant analysis model trained using the gut microbiota of individuals from China, India and Indonesia (representation of Chinese, Indian and Malay ethnic group, respectively) showed a better-than-random performance in classifying Malaysian of Chinese descent, although the performance for Indian and Malay were modest (true prediction rate, Chinese=0.60, Indian=0.49, Malay=0.44). Separately, differential abundance analysis singled out *Ligilactobacillus* as being elevated in Indians. We postulate that despite the strong influence of geographical factors on the gut microbiota, cultural similarity due to a shared ethnic origin drives the presence of a shared gut microbiota composition. The interplay of these factors will likely depend on the circumstances of particular groups of migrants.

## Data Summary

All raw sequence data are available online. The R and Bash script utilized for the meta-analysis has been uploaded on https://github.com/jdwiyanto/. The authors confirmed that all supporting data, code and protocols have been provided within the article or through supplementary data files.

**Impact Statement**

Current studies investigating the influence of ethnicity on gut microbiota are limited in their scope and size, hampering their interpretability. This meta-analysis obtained gut microbiota data from four countries that represent three ethnic groups: Chinese, Indian and Malay. Our result indicates a considerable overlap in the gut microbiota of individuals from the four distinct countries and observed the presence of a shared gut microbiota composition among ethnically similar individuals, despite geographically separation. Through this meta-analysis, we demonstrate the importance of the ethnic origin of an individual in influencing the gut microbiota.

## Introduction

Our understanding of the role of human gut microbiota in health and diseases has increased significantly over the past two decades [1–3]. This development has opened up the potential to modulate the gut microbiota to improve human health [4]. For instance, faecal transplantation is an effective treatment for *Clostridium difficile* infection [5]. However, due to the plasticity of the human gut microbiota, the links between disease and the compositional microbiota changes are often complicated [6]. This challenge highlights the need for a comprehensive understanding of the confounding factors that drive gut microbiota variation to accurately distinguish clinically irrelevant ‘noise’ from dysbiosis, i.e. the perturbation of the healthy gut microbiota [7].

Ethnicity has long been identified as a potential confounder of the gut microbiome [8, 9]. However, most available studies have focused on the Western setting, in which the level of gut microbial assimilation was evaluated (for example, Vangay *et al*. [10] on Hmong and Karen migration to the USA, Peters *et al*. [11] on the acculturation of Korean Americans, and Deschasaux *et al*. [12] on the gut microbiota of migrant communities in the Netherlands). These studies established that incomplete acculturation is the primary driver of gut-ethnicity variation in the Western communities, with the later generations of immigrants exhibiting largely overlapping gut microbiota profiles with their Caucasian counterpart. Brooks *et al*. [13] also reported gut microbiome variations across the four ethnic groups in the USA, although this observation was not strictly controlled for geographical variation.

In comparison, studies outside of these Western settings are scarce. Among those available, socioeconomic variation seemed to be the main determining factor for gut microbial composition. Chong *et al*. [14] postulated that unequal socioeconomic standing drove the gut microbiota variation across ethnicity in a community in Malaysia, suggesting that gut-ethnic association is multifaceted. We recently reported gut microbiota variation in a multiethnic Malaysian community with a relatively equal socioeconomic status [15]. We postulated that even in a multiethnic community with a long history of cohabitation, complete cultural assimilation might be hampered by innate cultural barriers between different ethnic groups, resulting in distinct gut microbiota. However, the previous study was conducted on a relatively small and geographically restricted population. In this study, we conducted a meta-analysis on 16S rRNA gene amplicons available in the public domain. Specifically, 16S rRNA gene amplicons from China, India, Indonesia and Malaysia were selected to evaluate the effect of Chinese, Malay and Indian ancestry and geographical separation on gut microbial composition.

## Methodology

### Identification of eligible research articles

This meta-analysis was prepared according to the preferred reporting items for systematic reviews and meta-analyses (PRISMA) [16], with the detailed list included as Text S1, available in the online version of this article. Briefly, a literature search strategy was employed on 6 July 2020 in the Scopus database to filter for human gut microbiome studies involving Chinese, Indian, Indonesian or Malay individuals. Only data from independent human subjects were included in the meta-analysis. For individuals engaged in a longitudinal study, only the control/data prior to the study intervention were included. Additionally, individuals who were explicitly suffering from an underlying disease or were clinical patients were excluded. A total of 375 articles were screened, and 112 remained after abstract filtering. We also included our study on a multiethnic Malaysian community, which comprised 175 individuals of Chinese, Indian or Malay descent [15]. After the final screening, a total of 16 studies were included in the final dataset.

### Raw read sequence extraction, filtering and processing

**Fig. 1.**
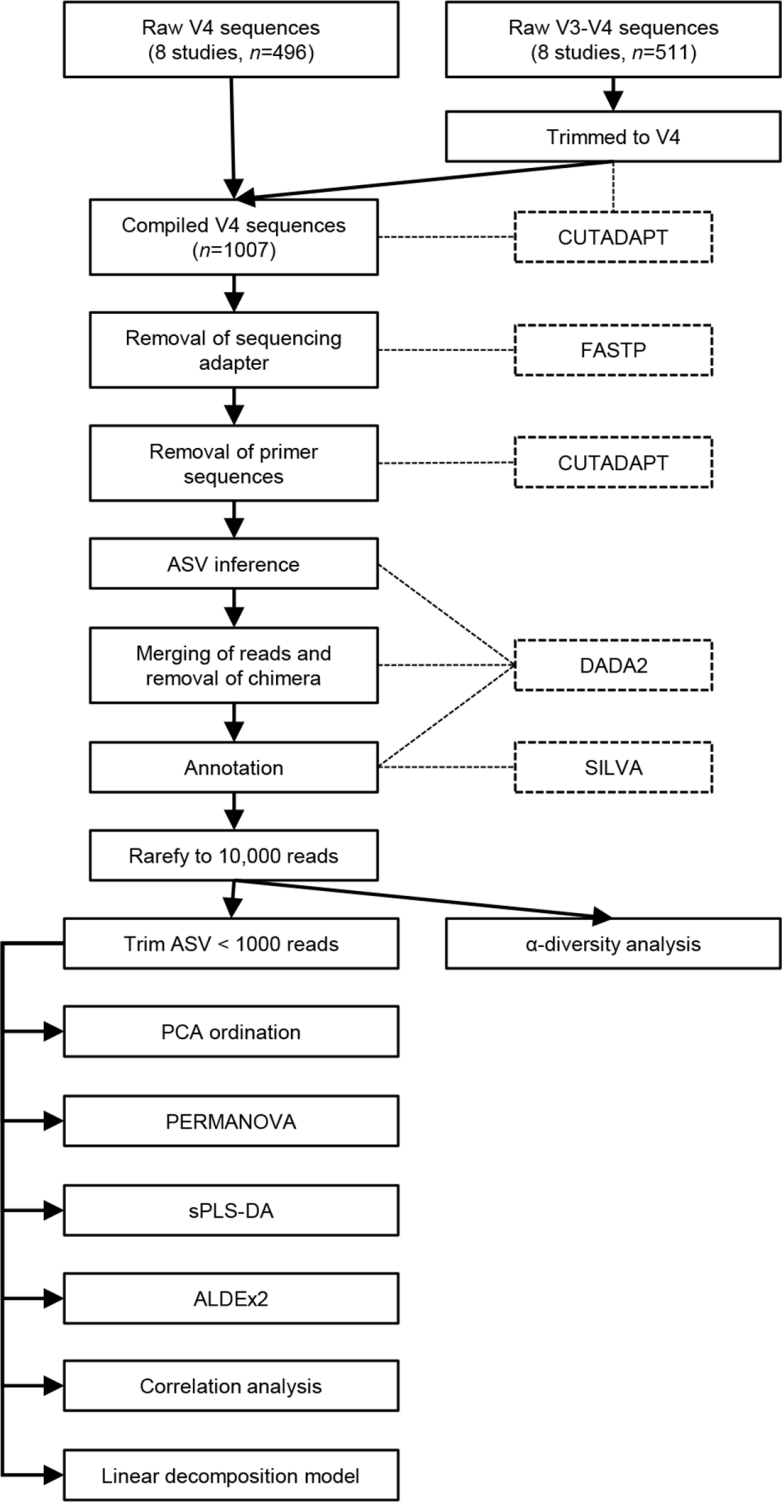
Overview of the analysis pipeline utilised in this meta-analysis.

An overview of the analysis pipeline utilized in this meta-analysis was visualized in Fig. 1. Publicly available raw sequence data that were sequenced on an Illumina platform and covered the V4 region of the 16S rRNA gene were included and extracted from either NCBI Sequence Read Archive (SRA) or European Nucleotide Archive (ENA) using SRA Toolkit (available at https://github.com/ncbi/sra-tools).

All raw sequences were pre-processed with fastp version 0.20.1 [17] to remove sequencing adapters. Subsequently, primer sequences were removed, and the biological sequences were trimmed to the V4 region based on the 515F and 806R primers using Cutadapt version 1.18 [18]. DADA2 version 1.16.0 [19] was then employed for Amplicon Sequence Variants (ASV) inference, merging paired-end reads, and chimera removal using the consensus method. The processed samples yielded a total of 53 063 786 sequences (mean 52 695±40 182 reads per sample). All merged sequences were confirmed to be from the V4 region based on their length (~252 bp) before sequence annotation using the assignTaxonomy function in DADA2 against the silva database version 138.1 [20].

Before the analysis, the dataset was agglomerated to the genus level to reduce inter-study variability and rarefied to 10 000 read depths using the function rarefy_even_depth in phyloseq version 1.32.0 [21], which was sufficient to capture most bacterial taxa (Fig. S1).

### α-diversity analyses

The α-diversity was inferred based on Chao1 and Shannon diversity indices. Chao1 measure the overall bacterial richness of the dataset and considers the presence of singletons and double counts to estimate the rare sequence variants, which might not be captured due to differing sequence read depth, while Shannon diversity index considers the richness as well as the evenness of the taxa in the dataset [22]. The α-diversity values were then estimated using the wrapper package phyloseq version 1.32.0 [21]. Differences between the communities were statistically compared using Kruskal–Wallis test, with post hoc Mann–Whitney U test and Benjamini–Hochberg correction wrapped under the package ggpubr version 0.4.0 [23].

### Ordination analyses

The abundance data were first filtered to exclude edges with less than 1000 raw counts. The centred-log ratio transformation under the propr package version 4.2.6 [24] was then applied, transforming the compositional nature of next-generation sequencing data into a simplex, enabling analysis in the Euclidean space. The transformed data were then ordinated using principal component analysis wrapped under phyloseq version 1.32.0 [21].

### Permutational multivariate analysis of variance (PERMANOVA)

PERMANOVA with 999 permutations was performed using the function adonis in the R package vegan version 2.5–6 [25] and adjusted for country, original 16S rRNA gene amplicon region, extraction kit and preservatives.

### Supervised analysis using sparse partial least squares – discriminant analysis

Sparse PLS-DA (sPLS-DA) models were run to classify participants based on their gut microbiota profile using the R package mixOmics version 6.12.2 [26]. sPLS-DA included a Lasso penalisation feature, which improved the classification performance for multiclass feature selection in high-throughput sequencing dataset [27]. The sPLS-DA model was trained using participants from China, India and Indonesia to classify their ethnicity as either Chinese, Indian or Malay (*n*=643). The model was validated by its ability to accurately classify the ethnicity of participants from a multiethnic Malaysian community (*n*=175) based on the model trained using the mainland subjects. The optimal number of components was determined through the perf function in the mixOmics package, with fivefold cross-validation and 50 repeats. The taxa that best differentiated the groups under the model was identified using the plotLoadings function in the mixOmics package.

### Differential abundance analysis using ALDEx2

Differential abundance analysis was conducted in ALDEx2 version 1.20.0 [28] using the generalized linear model in the function aldex.glm. The analysis was performed with 256 permutations using Monte Carlo simulation, and was controlled for the following variables: country, original 16S rRNA gene amplicon region, extraction kit and preservatives. Multigroup comparison in the ALDEx2 model was corrected using the Benjamini–Hochberg method.

### Heatmap and correlation plot analyses

Heatmap of the included studies and their metadata was generated using the R package ComplexHeatmap version 2.4.3 [29]. Taxa-ethnic Spearman correlation analysis was conducted using the R package corrplot version 0.84 [30], with an asterisk denoting a significant association (*P*<0.05). The Spearman partial correlation between taxa and ethnicity was analysed using the R package ppcor version 1.1 [31] and was adjusted for the following variables: country, original 16S rRNA gene amplicon region, extraction kit and preservatives.

### Linear decomposition model

A linear decomposition model (LDM) was run to identify taxa whose abundances were significantly different across the ethnic groups, using the R package LDM version 2.1 [32]. The analysis was run to classify the gut microbiota across ethnicity after accounting for country, extraction kit, original 16S rRNA gene amplicon region and preservatives. Default parameters were used for the test, and multigroup comparison was corrected using the Benjamini–Hochberg method with a 95 % confidence limit.

## Results

### Overview

A total of 16 studies comprised of 1007 individuals including Chinese (*n*=636), Indian (*n*=248) and Malay (*n*=123) ancestry were included in this meta-analysis (Tables 1 and S1) [33–47]. A relatively balanced number of studies utilized the V3-V4 and V4 region, and most of these studies utilised a Qiagen-based DNA extraction kit (Fig. S2). Most (*n*=12/16) of the studies did not use a preservation solution.

**Table 1.** List of gut microbiota studies involving Chinese, Indian or Malay communities included in this meta-analysis

Author	BioProject	No. sample	Country	Reference
Parker *et al*. 2017	PRJEB20773	40	India	[39]
Khine *et al*. 2020	PRJEB34323	63	Indonesia	[36]
Yin *et al*. 2017	PRJNA338148	13	China	[45]
Winglee *et al*. 2017	PRJNA349463	40	China	[44]
Schneider *et al*. 2017	PRJNA353065	8	Indonesia	[40]
Bian *et al*. 2017	PRJNA385551	300	China	[33]
Weng *et al*. 2019	PRJNA431126	24	China	[43]
Gaike *et al*. 2020	PRJNA448494	20	India	[35]
Duan *et al*. 2020	PRJNA480923	7	China	[34]
Zhou *et al*. 2020	PRJNA513244	69	China	[47]
Lappan *et al*. 2019	PRJNA525566	23	India	[38]
Kumbhare *et al*. 2020	PRJNA527121	10	India	[37]
Tang *et al*. 2019	PRJNA553183	100	India	[42]
Sun *et al*. 2019	PRJNA574565	60	China	[41]
Zeng *et al*. 2020	PRJNA578008	55	China	[46]
Dwiyanto *et al*. 2020	PRJNA631204	175	Malaysia	[15]

A large portion of the participants were from China (*n*=568), followed by India (*n*=193), Malaysia (*n*=175) and Indonesia (*n*=71). Out of 1007 individuals, 669 were explicitly stated as healthy and had no underlying diseases. The included participants ranged from 10 to 100 years old. There were more females among the included participants (449 females versus 219 males), although sex information was unavailable for two studies (detailed in Table S1).

### α-diversity analysis

**Fig. 2.**
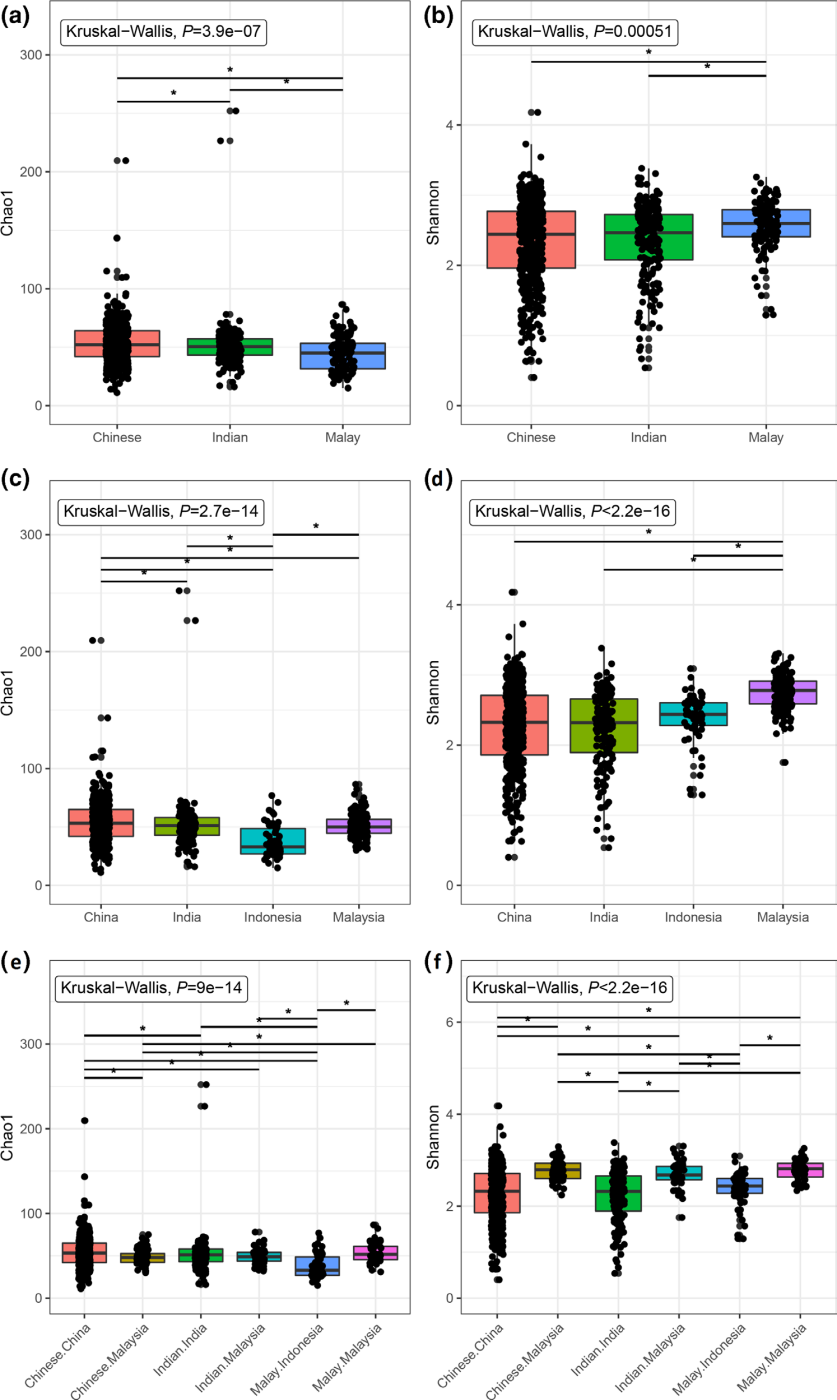
α-diversity estimates of Chinese, Indian and Malay communities based on Chao1 (a, c, e) and Shannon (b, d, f) index, classified according to ethnicity (top row), country or origin (middle row) or both (bottom row). Significance label indicates *q*<0.05 based on Mann–Whitney U test with the Benjamini–Hochberg correction.

Two measures were used for the α-diversity assessment. A significant difference in Chao1 richness profile between the communities was observed, with Indonesians exhibiting a significantly lower Chao1 index compared to all other groups (*q*<0.05, Fig. 2a, c and e). Comparatively, Malaysians had a significantly higher Shannon diversity compared to the other countries, regardless of their ethnicity (*q*<0.05, Fig. 2b, d and f).

### Ethnicity was significantly associated with the gut microbiota

**Fig. 3.**
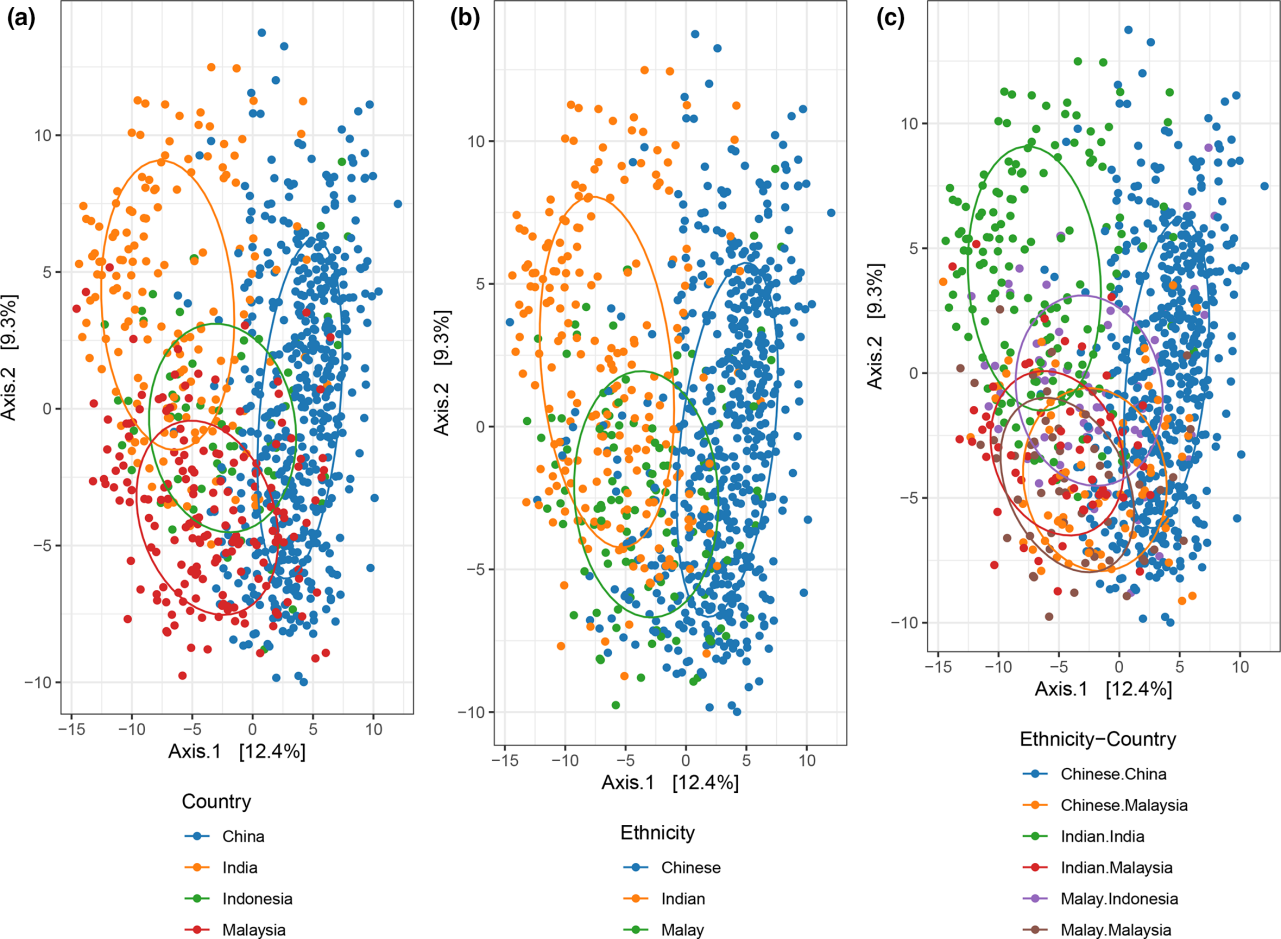
Principal component analysis based on centred log ratio-transformed dataset of the Chinese, Indian and Malay communities classified according to their country of origin (PERMANOVA, *R* ^2^=0.03, pseudo-*F*=14.14, *P*<0.05) (a), ethnicity (PERMANOVA, *R* ^2^=0.005, pseudo-*F*=2.643, *P*<0.05) (b), or both (PERMANOVA, *R* ^2^=0.04, pseudo-*F*=12.33, *P*<0.05) (c). All PERMANOVA analysis was adjusted for extraction kit, 16S rRNA gene amplicon region, and use of preservatives. Ellipses were based on a 60 % confidence level.

Gut microbiota of individuals from China and India formed two major clusters of the principal component analysis (Fig. 3a). Interestingly, Malaysia and Indonesia completely overlapped with China and India despite being geographically separated. Similarly, separation across ethnicity was observed (Fig. 3b), although these became less apparent after accounting for the country of origin (Fig. 3c). Despite this, Malaysian Chinese and Indian clustered more closely with China and India, respectively. On the other hand, Malays did not exhibit a clear clustering pattern, fully overlapping with the China and India clusters. Additionally, ordination profiles classified according to the original 16S rRNA gene amplicon region, extraction kit and preservatives were randomly distributed along the axes, suggesting these were not major confounders of the observed ethnic-geographical separation (Fig. S3). After adjusting for the country along with other possible confounders (original 16S rRNA gene amplicon region, extraction kit and preservatives), the PERMANOVA found ethnicity to be significantly associated with the gut microbiota (PERMANOVA *R* ^2^=0.005, pseudo-*F*=2.643, *P*=0.001).

### Gut microbiota predicted the ethnicity of Malaysians with a better-than-random performance

**Fig. 4.**
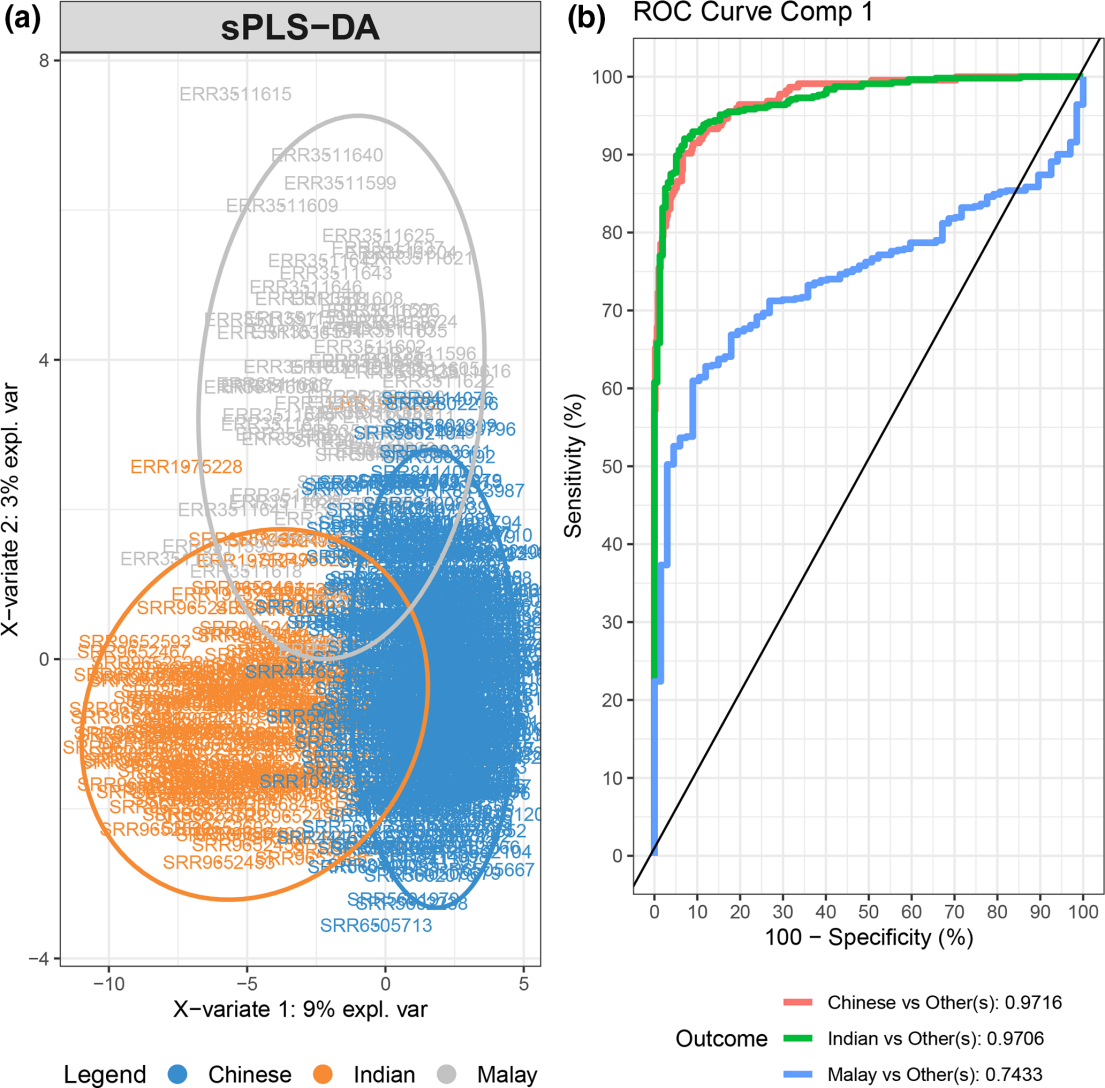
Sparse partial least squares – discriminant analysis (sPLS-DA) model trained using individuals from China, India and Indonesia, classified based on ethnicity (a) and the associated receiver operating characteristic (ROC) curve ().

We trained an sPLS-DA model to investigate whether the ethnicity could be accurately classified based on the gut microbiota despite geographical variation. A training model was trained using individuals from China, India and Indonesia to represent the Chinese, Indian and Malay ethnic groups, respectively (Fig. 4a). The model was assessed based on its accuracy in predicting the ethnicity of individuals from a multiethnic Malaysian community. This model distinguished individuals from China, India and Indonesia with receiver operating characteristics (ROC) curve showing an area under curve (AUC) of 0.97, 0.97 and 0.74, respectively (Fig. 4b). Importantly, the model performed with a better-than-random performance in classifying the ethnicity of Malaysian Chinese, with a more modest performance in classifying Indian and Malay (true prediction rate=0.60, 0.49 and 0.44, for Chinese, Indian and Malay, respectively, Table S2).

### Differential abundance analysis revealed ethnicity-associated taxa

**Fig. 5.**
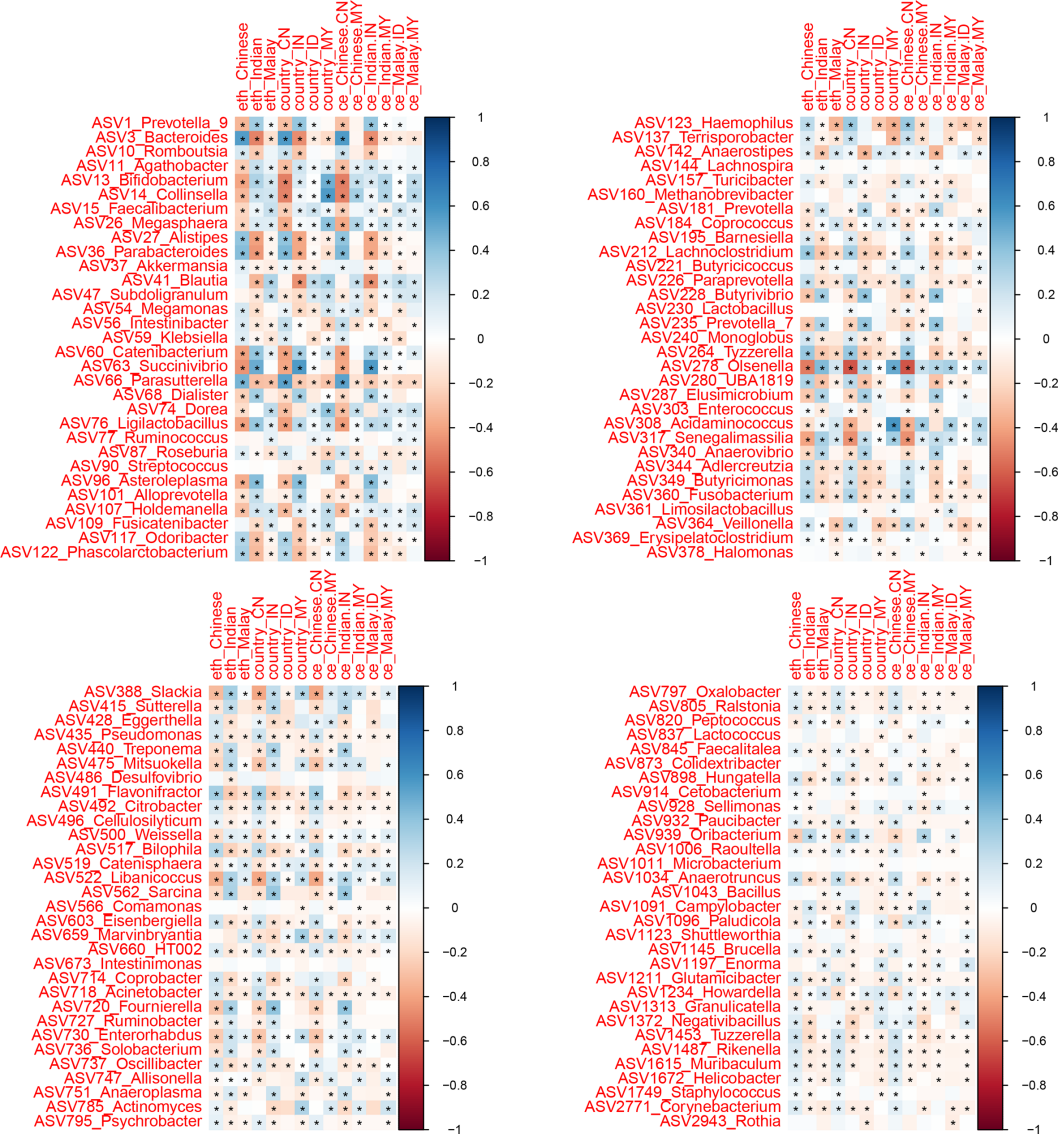
Spearman correlation plot between gut microbiota taxa and country-ethnicity variables. Asterisk indicates a significant association within a 95 % confidence interval.

We investigated further by correlating the observed taxa with ethnicity, country and ethnicity-country (Fig. 5). Most of the taxa recorded a significant association (*P*<0.05) with either of the tested variables, even after controlling for possible confounders (Table S3). A multivariate model comparing the three ethnic groups were performed using LDM, which identified eight taxa significantly associated with ethnicity (*q*<0.05, Fig. S4, Table S4). Notably, *Ligilactobacillus* and *Bifidobacterium* were elevated in Indian when the comparison was made with Chinese. However, ALDEx2 analysis found only *Ligilactobacillus* to be significantly associated with ethnicity, being elevated in Indian compared to the other two ethnic groups (ALDEx2, estimate: 3.84, SE: 0.82, *q*<0.05).

## Discussion

This meta-analysis provides novel insights into how ethnicity modulates the gut microbiota. Specifically, we observed a shared gut identity across ethnically similar communities from different geographical regions. Importantly, these shared traits enabled the classification of Malaysians of Chinese and Indian descent based on their mainland counterparts’ gut profiles with modest success.

No discernible effect of ethnicity on α-diversity was observed, suggesting that α-diversity was driven mainly by regional variation. The higher α-diversity measures observed among Malaysians on average, as reflected by the Shannon measure, likely reflected the multiculturalism practised in the country [43], exposing the community to a broader range of environmental variables (e.g. food choices). Besides, α-diversity likely reflected the different urbanization levels of the studied communities. The negative impact of urbanization on gut bacterial diversity has been reported [48, 49], although modernization has also been positively associated with gut diversity [44]. Nevertheless, the higher heterogeneity observed in China and India’s microbiota might be due to the inclusion of multiple communities spanning a broad urbanization spectrum.

The observed overlap of gut profiles from the Southeast Asian communities with India and China possibly reflects the strong cultural influence that country of ethnic origin exerts on these communities, showing that gut microbiota variation does not necessarily correlate with geographical distance. The Chinese community in Malaysia can be traced back to mass migration events, mainly from southern China [50]. Similarly, Malaysian Indians could be traced back to the mass migrations of south Indians during the colonization period in the early nineteenth century [51, 52]. Likewise, Malay culture was also heavily influenced by India [53].

Ethnically similar communities possibly share similar cultural practices due to their heritage, leading to similar lifestyles despite geographical separation. This cultural contrast stands true even in a multicultural society such as Malaysia. Lee [54] has previously argued that each ethnic group’s distinct heritage guides their dietary preference in Malaysia despite other culture-specific cuisines brought forth by the multicultural Malaysian society. In this regard, the higher abundance of *Ligilactobacillus* in Indians might reflect their affinity to dairy products, a common Indian diet ingredient [55]. This finding suggests that dietary differences across ethnic groups might be responsible for driving the observed gut-ethnic variations, which agreed with our previous study [15]. Nevertheless, dietary data were not always available from gut microbiota studies, hampering efforts to elucidate the actual effects of diet in driving gut-ethnic variation, including in this meta-analysis. Additionally, the influence of genetics on the gut microbiota has also been reported [56, 57], although its influence was likely minor [58].

Unsurprisingly, ethnic differences are diluted in a migrant community compared to their mainland counterpart, likely due to assimilation into a multicultural society [11]. Nevertheless, the significant gut microbiota variation across ethnicity indicates that complete assimilation might not be achieved even after years of cohabitation, at least in a middle-income setting.

Interestingly, the outcome of this meta-analysis concurred with a recent Singaporean study that reported on the presence of gut microbiota variation across infants of different ancestry [59]. They found a higher abundance of *Bacteroides* and *Bifidobacterium* in pre-weaning Chinese and Indian infants, respectively. The authors speculated that the difference in the infants microbiota might be attributed to the infant’s exposure to their culture-specific diet through their mother’s breast milk. Although the root cause of this variation was not further explored, it supported our postulation on the distinct cultural practices driving gut microbiota variation in a multicultural society. Unfortunately, we were not able to obtain any gut microbiota sequence from a Singaporean community, which could support this notion.

Ussar and colleagues have previously reported on the persistence of gut microbiota variation on genetically similar mice sourced from different vendors, representing different environmental exposures [60]. Although the mice exhibited a largely similar microbiota after three generations of institutionalization, significant variation in their gut microbiota persisted. This observation opens up new possibilities in the factors driving common traits across the geographically distinct yet ethnically-similar communities, where a shared origin could have caused similar gut profiles despite having been segregated for generations.

It is worth noting that Khine *et al*. [61] had previously discounted ethnicity’s impact in favour of dietary preference in driving gut microbiota variation between Chinese children in Malaysia and China. Crucially, this study only focused on the differences across the ethnic groups and did not analyse shared gut microbiota traits across ethnically similar individuals. However, a closer look into the study observed an overlap in the ordination plot between Chinese in China and Malaysia, qualitatively supporting the outcome of this meta-analysis.

Recently, a Singaporean cohort also reported the lack of a gut-ethnic signature and the absence of unique dietary patterns across ethnicity. However, it is worth noting that most of their participants were of Chinese descent (61/75). Nevertheless, their study gave rise to an essential notion of the impact of urbanization on the gut-ethnic axis. Unfortunately, raw sequence data from these studies were unavailable to validate this hypothesis, and information regarding urbanisation level from gut microbiota studies was scarce, which we highlight as a limitation of this study.

The Malays comprised a diverse range of ethnic backgrounds in Southeast Asia, ranging from the Javanese to the western Indonesian Malay [62], with some recorded mass migration events of Malays from Indonesia to the Peninsular Malaysia late nineteenth century [63]. In Malaysia, the Malays classification is widely used as an umbrella term to unify individuals adhering to the official national religion, clouding its adherents’ genetic and ethnic background. The absence of a gut profile linking the Malaysian and Indonesian Malays likely reflected this situation, suggesting the Malays from the two nations were ethnically distinct and did not substantially share cultural practices.

By including a comprehensive list of publicly available gut microbiota sequences from India and China, this meta-analysis was robust against regional gut microbiota variations, a potential confounder in gut microbiota studies [64]. Moreover, explicitly diseased patients were excluded, ensuring that the observed variations were not due to drug intake or disease [65, 66]. Despite this, the limited number of gut microbiota studies from the southeast Asian region and Malaysia, in particular, limited the interpretability of this study. Also, the scarcity of studies involving immigrant Chinese and Indian communities in the western setting represented another challenge in confirming our hypothesis. This limitation was further compounded by the limited accessibility of raw research data, a known barrier to a comprehensive comparative analysis of gut microbiota studies [67]. Additionally, information on the socioeconomic [14] and urbanization level [48] of the participants was largely unavailable, which could have influenced the outcome of this meta-analysis. Nevertheless, the result of this meta-analysis is in agreement with our hypothesis that the long-term effect of ethnicity-driven cultural practices modulates the gut microbiota in the absence of recent migration events and socioeconomic disparity [15]. Indeed, cultural variation is a strong determinant of dietary choices. In Malaysia, individuals of Chinese descent reported the highest consumption of animal protein in general and pork specifically, while beef consumption was most frequently reported by the Malays [68]. In contrast, ethnic Indian consumed the least animal protein and the most plant protein [68]. Similarly, mainland Indians mostly consumed a cereal-based diet with low consumption of animal proteins [69]. Furthermore, the unique herbs and spices utilized in different cuisines further distinguish each ethnicity’s dietary pattern. For example, star anise [70] and Sichuan pepper [71] are common ingredients in Chinese cuisine but less common in others. Lastly, the relatively small effect of ethnicity on Malaysians’ gut microbiota in terms of the overall microbiota composition is not surprising given the duration of cohabitation. Nevertheless, the differential abundance of specific taxa might indicate certain ethnic groups’ adherence to a particular lifestyle and dietary practices.

## Conclusion

Persistent cultural preference will inherently result in gut microbiota variation in a multiethnic society. We highlight the importance of accounting for ethnicity, even in studies involving communities with a long history of cohabitation.

## Supplementary Data

Supplementary material 1

Supplementary material 2
