# Supplementary material for: Geographical separation and ethnic origin influence the human gut microbial composition: a meta-analysis from a Malaysian perspective
Source: Microb Genom. 2021 Aug 31;7(8):000619. doi: 10.1099/mgen.0.000619 (PMC8549367; doi:10.1099/mgen.0.000619)

Supplementary Text and Figures for:

Geographical separation and ethnic origins influence the human gut microbial composition: A meta-analysis from a Malaysian perspective

Jacky Dwiyanto[1], Qasim Ayub[1,2], Sui Mae Lee[1], Su Chern Foo[1], Chun Wie Chong[3,4], Sadequr Rahman[1,5]

[1] School of Science, Monash University Malaysia, Bandar Sunway, Malaysia

[2] Monash University Malaysia Genomics Facility, Bandar Sunway, Malaysia

[3] School of Pharmacy, Monash University Malaysia, Bandar Sunway, Malaysia

[4] Institute for Research, Development and Innovation, International Medical University, Kuala Lumpur, Malaysia

[5] Tropical Medicine and Biology Multidisciplinary Platform, Monash University Malaysia, Bandar Sunway, Malaysia

Corresponding author:

Jacky Dwiyanto, email: jacky.dwiyanto@monash.edu

Sadequr Rahman, email: sadequr.rahman@monash.edu

Captions

Supplementary Text S1. Preferred Reporting Items for Systematic Reviews and Meta-Analyses (PRISMA) – methodology

Supplementary Figure S1. Rarefaction curve based on samples with less than 30,000 read depths, showing near saturation of the number of detected taxa at the 10,000 read depths point

Supplementary Figure S2. Distribution of the country, extraction kit, 16S region, and preservatives used in the studies included in this meta-analysis

Supplementary Figure S3. Principal component analysis based on centred log ratio-transformed dataset of the Chinese, Indian and Malay communities classified according to their country of origin (a), ethnicity (b), extraction kit (c), original 16S region (d), and preservatives (e)

Supplementary Figure S4. Relative abundance of taxa significantly associated with ethnicity analysed using the Linear Decomposition Model ($q<0.05$)

Supplementary Table S1. A detailed list of the studies included in this meta-analysis, including extraction methods, preservative usage, target 16s rRNA region, inclusion and exclusion criteria, and filtering parameters applied for inclusion in this analysis

Supplementary Table S2. Confusion matrix of the sPLS-DA model trained using gut microbiota data from individuals from China, India, and Indonesia, in classifying the ethnicity of individuals from a Malaysian community

Supplementary Table S3. Partial Spearman correlation analysis between ethnicity and gut microbiota taxa

Supplementary Table S4. List of taxa associated with ethnicity based on the Linear Decomposition Model. The significant value for multiple group comparison was adjusted using the Benjamini-Hochberg method.

Supplementary File 1

Preferred Reporting Items for Systematic Reviews and Meta-Analyses (PRISMA) – methodology

Protocol and registration – the protocol for this meta-analysis has not been registered elsewhere.

Eligibility criteria – This meta-analysis imposed the following eligibility criteria: articles written in English; study samples originated from human faecal sample as a proxy for the distal colon; employed a 16S rRNA amplicon sequencing strategy on Illumina platform to minimise instrument bias; the amplified 16S rRNA region covers the V4 hypervariable region; study participants were sourced from either a health or community cohort and were generally healthy i.e. not suffering from any chronic diseases. Those with antibiotic consumption history were excluded when the information was available; study participants were 10 years or older, and data were publicly available in the public repository (NCBI or ENA).

Information sources – eligible articles were identified through utilisation of search strings in the SCOPUS database.

Search – Literature search was conducted using the following search strategy: title, abstract and keywords containing (“chinese*” OR “malay*” OR “Indonesia*” OR “india*”) AND ("gut" OR "f*cal" OR "gastro*" OR "intestin*" OR "stool*") AND ("microbiome" OR "microbiota"))) AND ("16s*" OR "amplicon*" OR "rRNA*") and excluded articles with the title, abstract or keywords containing ("animal" OR "mouse" OR "mice" OR "rat" OR "zebra*")) AND ("pyrosequencing" OR “mice”).

Study selection – The list of publications identified based on the above search criteria were manually screened based on their abstract and methodology. All studies which fulfilled the eligibility criteria were included in the meta-analysis

Data collection process – Accession number for each included study was obtained. The participants from each study was then screened using the available metadata either through the repository or from the study itself to exclude non-independent participants (e.g. longitudinal study) and those who did not meet the eligibility criteria (e.g. case participant in a case-control study). Sequencing primer information was also obtained in a similar manner for the removal of non-biological nucleotides from the raw sequences.

Data items – The following information was collected from each study: geographical location (which includes country and region) and ethnicity. Ethnicity was recorded based on the information provided by the original study. When this information was unavailable, the ethnicity was assumed based on location (e.g. Chinese Han for studies from China).

Risk of bias in individual studies – Different studies employed different health criteria, ranging from assumption of health to a more stringent criteria such as medical records. We did not standardise these criteria and employed 'healthy' participants according to the author's original criteria.

Some studies employed a longitudinal analysis, which could cause bias through non-independent samples. To minimise this bias, only independent participants were included. For case-control studies, only healthy controls were included.

Summary measures – The final output of the meta-analysis yielded gut microbiota abundance profile.

Synthesis of results – not applicable.

Risk of bias across studies – Bias risk from variations outside of geographical or ethnicity influence were minimised through the exclusive inclusion of healthy participants. Only studies with raw sequence data derived from the V4 hypervariable region of the 16S rRNA gene and sequenced on an Illumina platform instrument was used to minimise known sequence bias through variation in sequence target and instrument. The trimming of non-V4 16S rRNA regions was necessary to avoid comparing datasets with variable starting sequence position and lengths (e.g. comparing V3-V4 datasets with V4 datasets). An unequal sequence comparison might confound the analysis due to variations in binding efficiencies and amplification profiles from the different sequencing primers used [1]. All sequence data was processed and analysed using a single pipeline to minimise the bias introduced in different processing pipeline. Differences in DNA extraction and preservation methods were not controlled due to their vast variability, but were statistically adjusted for in the downstream analyses.

Additional analyses – not applicable.

References

[1]Bukin Y. S., Galachyants Y. P., Morozov I. V., Bukin S. V., Zakharenko A. S. et al. The effect of 16S rRNA region choice on bacterial community metabarcoding results. *Scientific Data*. **6(1)**, 190007; 10.1038/sdata.2019.7 (2019).

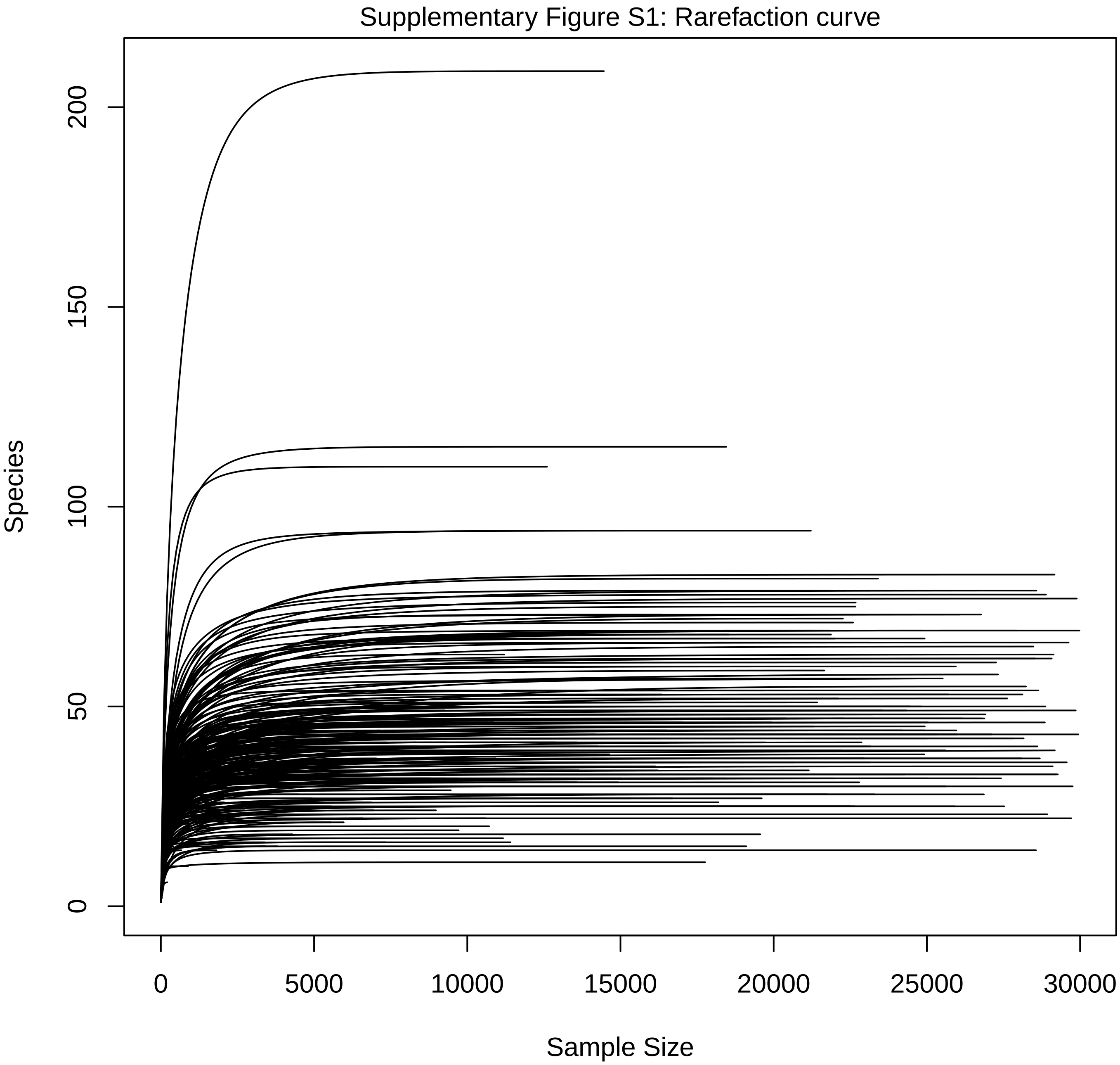
Supplementary Figure S1: Rarefaction curve
Species
0
50
100
150
200
Sample Size
0
5000
10000
15000
20000
25000
30000

Supplementary Figure S2: Heatmap of metadata

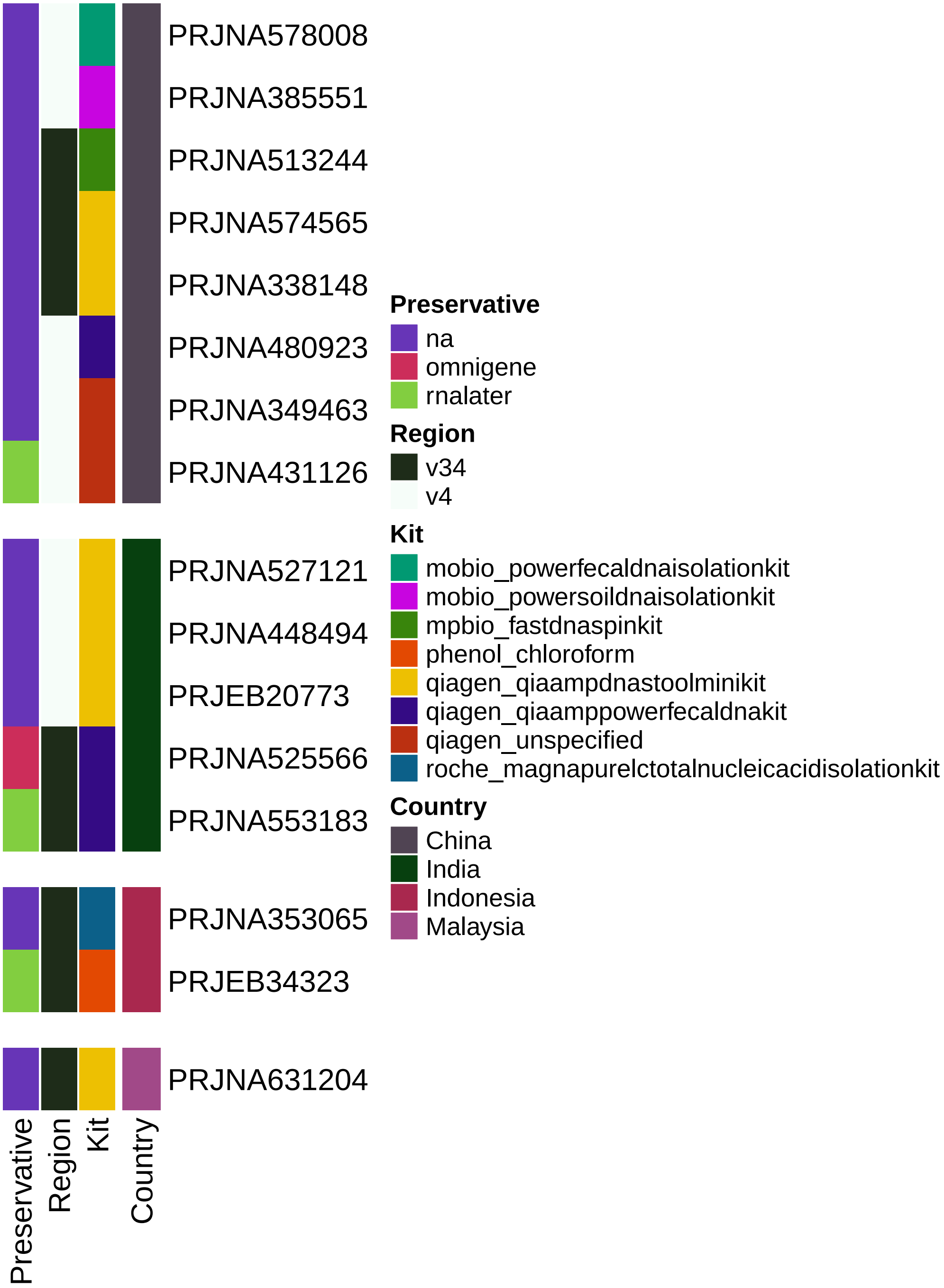

Supplementary Figure S3: Ordination plot of possible confounders

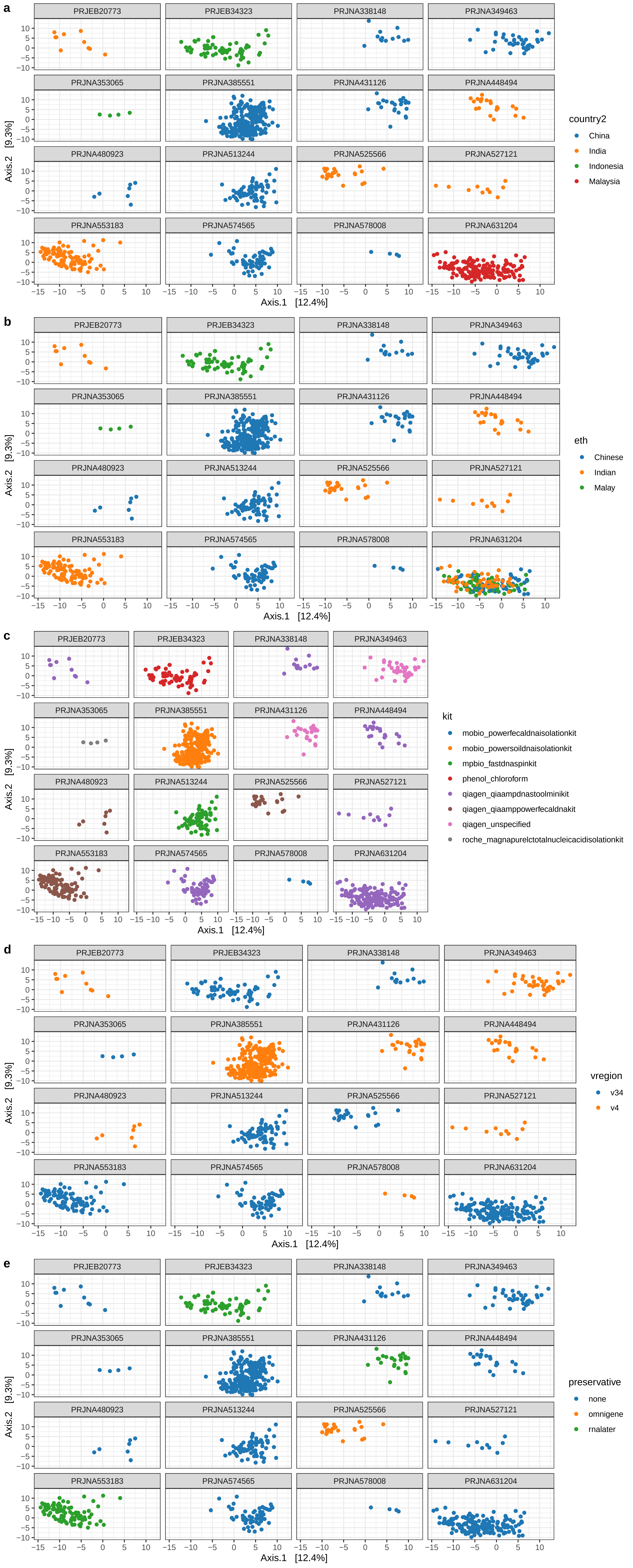

Supplementary Figure S4: LDM significant taxa

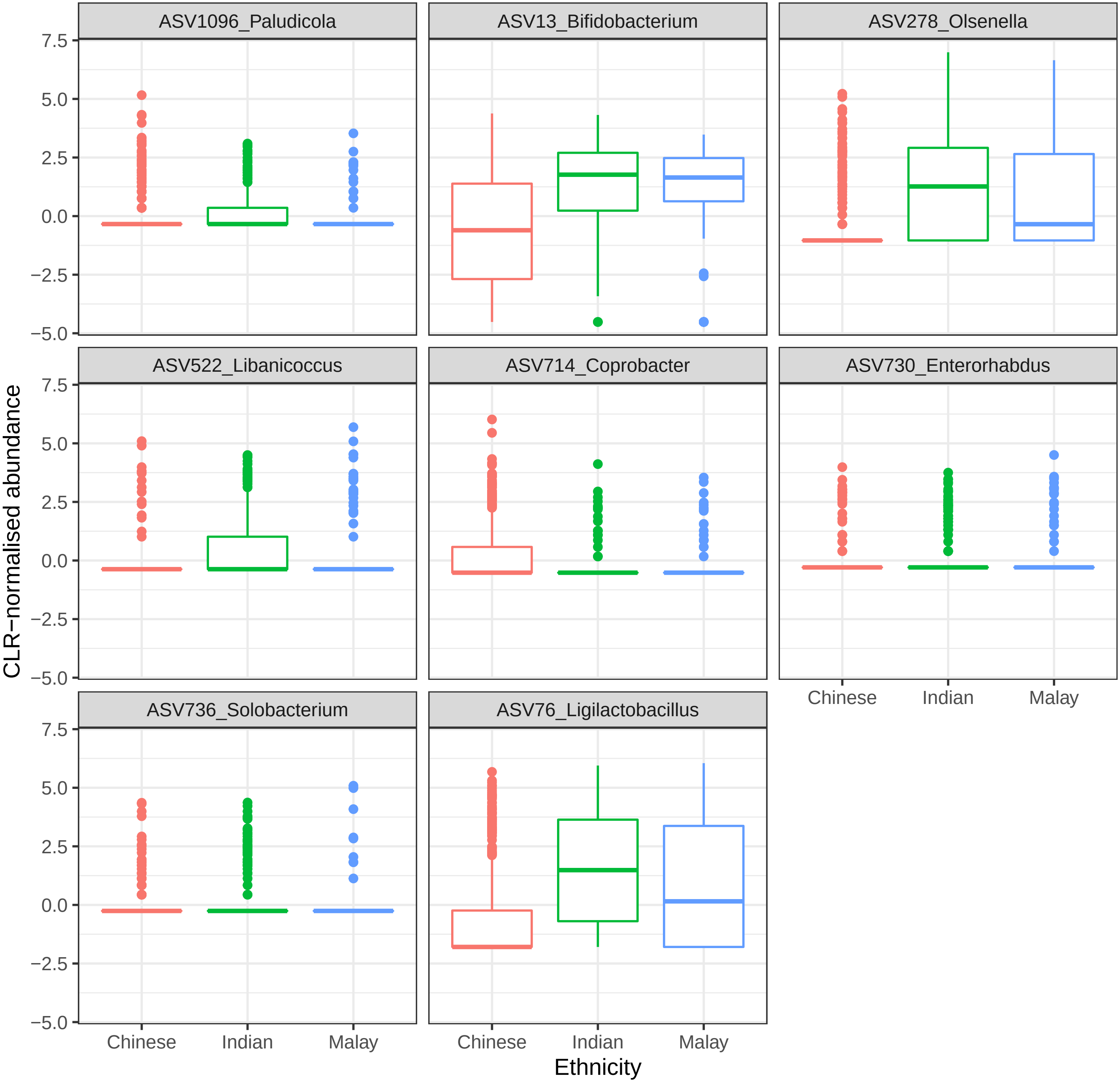

Supplement: Supplementary material 1 [file mgen-7-0619-s001.pdf]
